# Physical connections between different SSVEP neural networks

**DOI:** 10.1038/srep22801

**Published:** 2016-03-08

**Authors:** Zhenghua Wu

**Affiliations:** 1 School of Computer Science and Engineering, University of Electronic Science and Technology of China, ChengDu, 610054, China; 2 Key Laboratory for NeuroInformation of Ministry of Education, School of Life Science and Technology, University of Electronic Science and Technology of China, ChengDu, 610054, China

## Abstract

This work investigates the mechanism of the Steady-State Visual Evoked Potential (SSVEP). One theory suggests that different SSVEP neural networks exist whose strongest response are located in different frequency bands. This theory is based on the fact that there are similar SSVEP frequency-amplitude response curves in these bands. Previous studies that employed simultaneous stimuli of different frequencies illustrated that the distribution of these networks were similar, but did not discuss the physical connection between them. By comparing the SSVEP power and distribution under a single-eye stimulus and a simultaneous, dual-eye stimulus, this work demonstrates that the distributions of different SSVEP neural networks are similar to each other and that there should be physical overlapping between them. According to the band-pass filter theory in a signal transferring channel, which we propose in this work for the first time, there are different amounts of neurons that are involved under repetitive stimuli of different frequencies and that the response intensity of each neuron is similar to each other so that the total response (i.e., the SSVEP) that is observed from the scalp is different.

When a visual stimulus with a constant intensity and frequency presents in the visual field, the components of the EEG that share a similar frequency as the stimulus as well as its harmonics are strengthened^1^. This evoked EEG is called the Steady-State Visual Evoked Potential (SSVEP). The SSVEP has been observed in a wide frequency band from approximately 5 to 90 Hz^2^. The intensity of the SSVEP is closely related to the subjects themselves^2^,^3^, which suggests that the power of the SSVEP produced by the same stimulus could differ across subjects. The intensity of the SSVEP is also related to the modulation depth of the stimulus^2^ wherein a larger modulation depth produces a stronger SSVEP. Another important factor that is related to the SSVEP power is the stimulus frequency wherein the strongest SSVEP is located in the α band.

Since the discovery of the SSVEP, it has been used in many fields. One important use is to study the process of a recognition task over a long duration of time^4^,^5^,^6^,^7^. In this task, the disturbance between trials can be different, such that the superposition method that is often used for extracting ERP in a short duration cannot remove all disturbances and is not efficient in understanding the process of the task. Considering that parts of the SSVEP neural network can overlap with parts of the visual recognition network and that the SSVEP has a great anti-disturbance ability, some researchers believe that combining the SSVEP extraction method with the superposition method is a valid method for elucidating the process of a recognition task over a long duration of time^6^. This method is referred to as the Steady-State Probe Topography (SSPT) method. There have been many studies concerning the use of the SSPT method. These studies often include the activation of only one SSVEP network with only the connection between the one SSVEP network and the recognition network being discussed^8^. Another important use of the SSVEP method is to build an SSVEP-based Brain-Computer Interface (BCI)^9^,^10^,^11^,^12^,^13^. By utilizing different frequency flickers to represent different tasks, a subject can complete a task by simply staring at the flicker that corresponds to that work. By recognizing the SSVEP frequency in the evoked EEG, the intention of the subject can be decoded. This type of BCI has advantages, including a large level of anti-disturbance and a high transfer rate^7^,^14^,^15^,^16^. In SSVEP-based BCI, there are multiple SSVEP networks that are activated simultaneously; however, researchers have focused on recognizing the SSVEP frequency quickly and accurately, and none have yet discussed the connection between the SSVEP networks.

Although the SSVEP has been used widely, its mechanism is not yet clear. For a transient light stimulus, the transient response can be obtained by using the superposition method; however, for a continual flicker stimulus, the response is not equivalent to the product of the superposition of the transient responses. For a transient light stimulus, many neurons are involved in the response over a period of time. Because of different frequency characteristics of the axon hillock and its associated structures, this period of response is different. When another stimulus presents during this response period, the neuron cannot respond again. Therefore, the total response to serially repetitive stimuli, as observed from the scalp, is not the superposition of the transient response. In order to understand the mechanism of the SSVEP, many theories have been proposed. One theory suggests that the SSVEP is a cortical response that acts over the cortico-cortico loops in response to the stimulus that is presented to the peripheral retina. In this theory, three SSVEP networks exist that correspond to different frequency bands: low (5–12 Hz), medium (12–30 Hz) and high (30–50 Hz) frequency^2^. This theory follows from the observation that the SSVEP power does not increase or decrease linearly with an increase in stimulus frequency but rather there are three similar response curves in three nearby frequency bands. This theory is widely accepted in many SSVEP studies^17^,^18^,^19^. Some other studies have demonstrated that resonance exists in these frequency bands^12^,^16^.

It is presently unknown if these SSVEP networks do exist and if their physical distributions are the same or if there exists some physical overlapping between them. A few studies have made use of simultaneous flickers with different frequencies to study the different SSVEP networks^7^,^20^. In doing so, they have demonstrated that the distribution of SSVEP power was similar to each other. The SSVEP power under multiple flickers was smaller than that under only one flicker. This result demonstrates that the distribution of different SSVEP networks can be similar; however, because the spatial resolution of EEG is low, the distribution similarity between the SSVEP networks cannot demonstrate whether there is some overlapping between them. Conversely, the modulation depth of each stimulus following multiple flickers decreases when compared with only one flicker, because the SSVEP intensity is related to the modulation depth such that a decrease in SSVEP power can be caused by a decrease in modulation depth. However, this relationship is not guaranteed as parts of one SSVEP network can be shared by another SSVEP network. In other words, a decrease in the SSVEP power following multiple flickers cannot yet demonstrate whether there is some physical overlap between the different networks.

In this study, the SSVEP power and distribution following single-eye stimulation were obtained. Then, these parameters were obtained following dual-eye stimulation by flickers of different frequencies being simultaneously employed with no luminance influence between the flickers. Under stimulation in both eyes, the physical property of the stimulus for each eye is the same as that under the situation of a single-eye. Therefore, the SSVEP power and distribution differences between these two conditions can only be the result of the interaction between the SSVEP networks. The low and middle frequencies were selected as the stimuli in this study. The results show that the distribution of the different SSVEP networks is similar and that there exists some physical overlap between the different networks. Band-pass filter theory was first used to explain the signal transfer between the neurons. Based on this theory, there are different amounts of neurons involved under different frequency stimuli, so the SSVEP power varies across frequencies.

## Results

### The SSVEP effectiveness

Under every stimulus style, a clear SSVEP can be observed from the frequency spectrum. The ANOVA results demonstrated that significant differences exist between the evoked EEG and the spontaneous EEG in each situation. Here, we use the first stimulus style as an example. A stimulus of 12.5 Hz in only the left eye produced an ANOVA result of F(1,20) = 4.32 (p = 0.01) between the evoked condition and the spontaneous condition, which indicates that a power of 12.5 Hz in the evoked EEG is significantly larger than that in the spontaneous EEG. A stimulus of 16.7 Hz in only the right eye produced an ANOVA result of F(1,20) = 6.78 (p = 0.01), which indicates that a power of 16.7 Hz in the evoked EEG is significantly larger than that in the spontaneous EEG.

**Figure 1:**
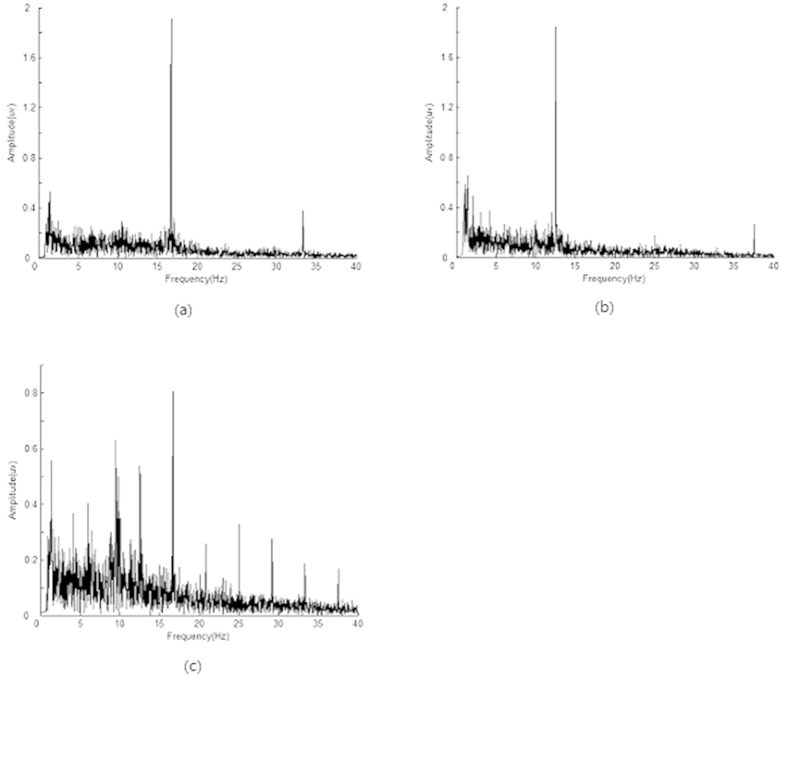
The frequency spectrum of SSVEP following varying stimuli for subject WLC at electrode No. 76. (**a**) for a stimulus of 16.7 Hz in the right eye alone, (**b**) for a stimulus of 12.5 Hz in the left eye alone, or (**c**) for a simultaneous stimulus of 12.5 Hz in the left eye and 16.7 Hz in the right eye.

A simultaneous stimulus of 12.5 Hz in the left eye and 16.7 Hz in the right eye produced an ANOVA result of F(1,20) = 1.8 (p = 0.04) and F(1,20) = 2.3 (p = 0.02), respectively. This indicates that a power of 12.5 Hz and 16.7 Hz in the evoked EEG are significantly larger than in the corresponding components in the spontaneous EEG. Following simultaneous stimulation, the evoked responses included not only the frequency components that were the same as the stimuli and its harmonics but also many other related components. Figure 1 shows the frequency spectrum of subject WLC at the occipital lobe electrode No. 76.

### The SSVEP power

Under the first style of stimulus, a power of 12.5 Hz SSVEP was observed to be larger than that of 16.7 Hz following a single eye stimulation of F(1,10) = 6.6, (p = 0.003). This was also observed when simultaneously stimulating the left eye with 12.5 Hz and the right eye with 16.7 Hz (F(1,10) = 6.08, p = 0.004). A power of 12.5 Hz SSVEP following single eye stimulation was significantly larger than following simultaneous stimulation (F(1,10) = 4.4, p = 0.01). A power of 16.7 Hz SSVEP following single eye stimulation was significantly larger than under the condition of simultaneous stimuli (F(1,10) = 8.4, p = 0).

Under the second style stimulus, there was no significant difference between an SSVEP power of 12.5 Hz and 8.3 Hz following single eye stimulation (F(1,10) = 2.0, p = 0.14). This was also observed when simultaneously stimulating the left eye with 12.5 Hz and the right eye with 8.3 Hz (F(1,10) = 2.4, p = 0.09). An SSVEP power of 12.5 Hz following a single eye stimulation was significantly larger than following simultaneous stimulation (F(1,10) = 5.8, p = 0). An SSVEP power of 8.3 Hz following single eye stimulation was significantly larger than that under the condition of simultaneous stimuli (F(1,10) = 3.4, p = 0.03).

Under the third style stimulus, there was no significant difference between an SSVEP power of 16.7 Hz and 6.3 Hz following single eye stimulation (F(1,10) = 1.8, p = 0.19). This was also observed when simultaneously stimulating the right eye with 16.7 Hz and the left eye with 6.3 Hz (F(1,10) = 1.3, p = 0.36). An SSVEP power of 16.7 Hz following single eye stimulation was significantly larger than under a condition of simultaneous stimuli (F(1,10) = 3.4, p = 0.03). An SSVEP power of 6.3 Hz following single eye stimulation was significantly larger than following simultaneous stimulation (F(1,10) = 3.9, p = 0.02). Table 1 shows the relative-power of the SSVEP and the ANOVA results under all conditions for different subjects, which are labeled ‘S1’, ‘S2’, …, and ‘S11’.

### The SSVEP distribution

Under the first style of stimulus, the SSVEP powers of 16.7 Hz and 12.5 Hz were concentrated in the occipital area. For different frequency stimulus, the SSVEP distribution was similar to each other. Following single-eye stimulation, the average correlation coefficient between the SSVEP distribution of 16.7 Hz and 12.5 Hz was 0.87. The ANOVA result was F(1,10) = 3.5 (p = 0.2), which indicated a distribution of 16.7 Hz was similar to that of 12.5 Hz. When simultaneously stimulating the right eye with 16.7 Hz and the left eye with 12.5 Hz, the average correlation coefficient between the SSVEP distribution was 0.85. The ANOVA result for the SSVEP distribution was F(1,10) = 2.1 (p = 0.18), thereby indicating a distribution of 16.7 Hz was similar to 12.5 Hz under the same condition.

**Figure 2:**
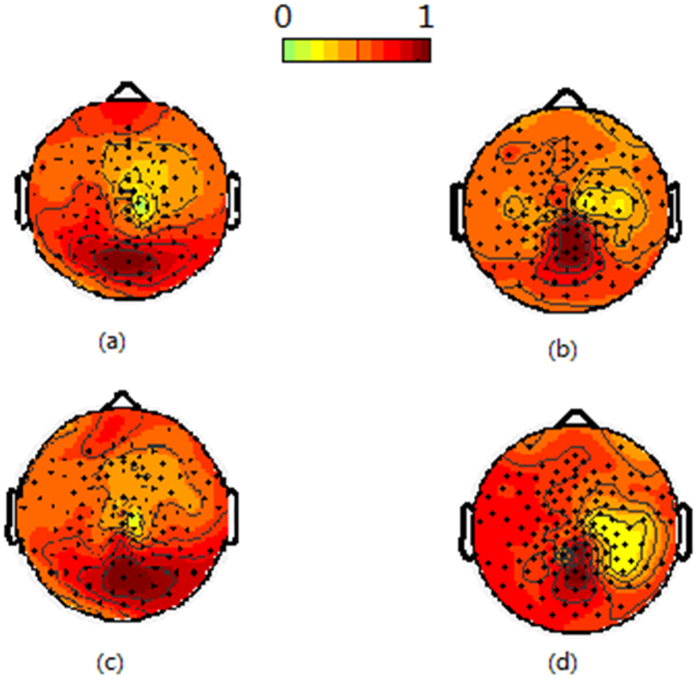
The average SSVEP distribution across all subjects following the first type of stimulus style. (**a**) indicates the 16.7 Hz distribution when only stimulating the right eye, (**b**) indicates the 12.5 Hz distribution when only stimulating the left eye, (**c**) indicates the 16.7 Hz distribution when stimulating the right eye with 16.7 Hz and the left eye with 12.5 Hz, and (**d**) indicates the 12.5 Hz distribution when stimulating the right eye with 16.7 Hz and the left eye with 12.5 Hz.

For the same frequency stimulus, the SSVEP distribution following single-eye stimulation was similar to that under the simultaneous stimuli also. For an SSVEP of 16.7 Hz, the ANOVA result for the SSVEP distribution was F(1,10) = 1.6 (p = 0.24). For an SSVEP of 12.5 Hz, the ANOVA result for the SSVEP distribution was F(1,10) = 2.1 (p = 0.22). Figure 2 shows the average SSVEP distribution across all subjects under the first stimulus style.

**Figure 3:**
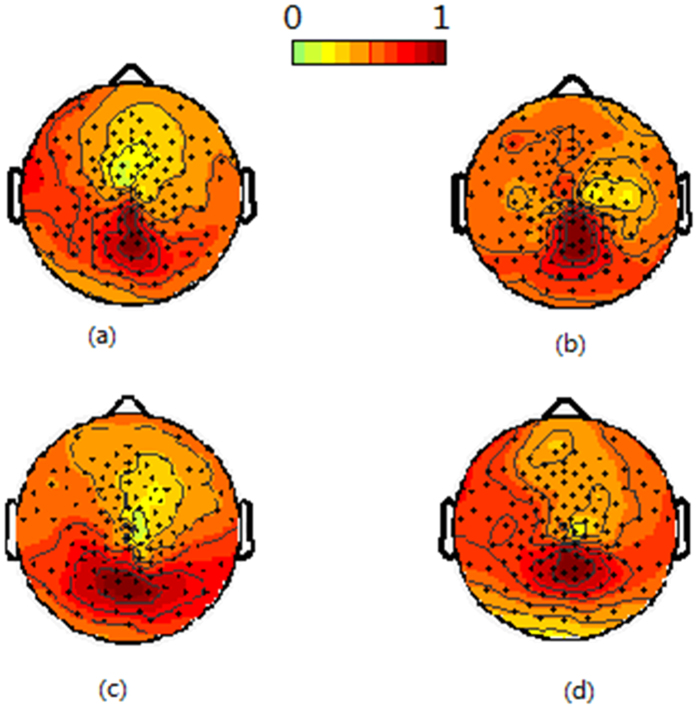
The average SSVEP distribution across all subjects under the second kind of stimulus style. (**a**) indicates the 8.3 Hz distribution when only stimulating the right eye, (**b**) indicates the 12.5 Hz distribution when only stimulating the left eye, (**c**) indicates the 8.3 Hz distribution when stimulating the right eye with 8.3 Hz and the left eye with 12.5 Hz, (**d**) indicates the 12.5 Hz distribution when stimulating the right eye with 8.3 Hz and the left eye with 12.5 Hz.

**Figure 4:**
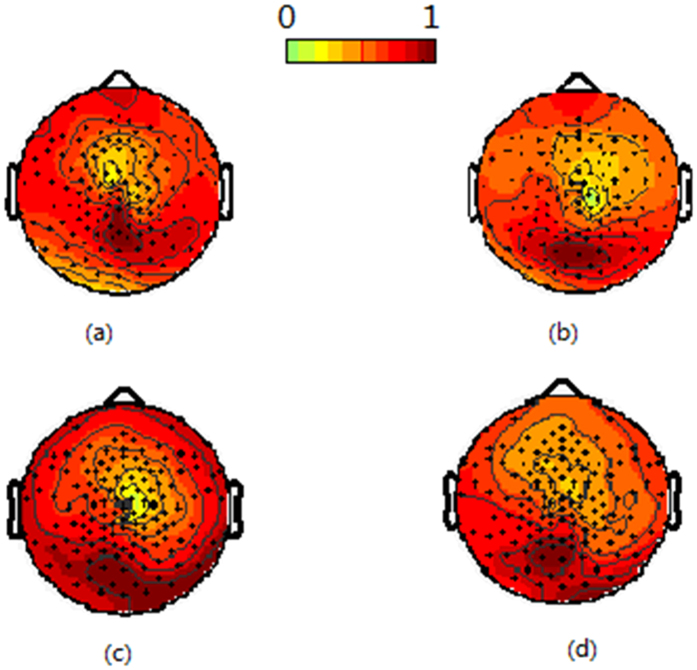
The average SSVEP distribution across all subjects under the second kind of stimulus style. (**a**) indicates the 6.3 Hz distribution when only stimulating the left eye, (**b**) indicates the 16.7 Hz distribution when only stimulating the right eye, (**c**) indicates the 6.3 Hz distribution when stimulating the right eye with 16.7 Hz and the left eye with 6.3 Hz, and (**d**) indicates the 16.7 Hz distribution when stimulating the right eye with 16.7 Hz and the left eye with 6.3 Hz.

**Figure 5:**
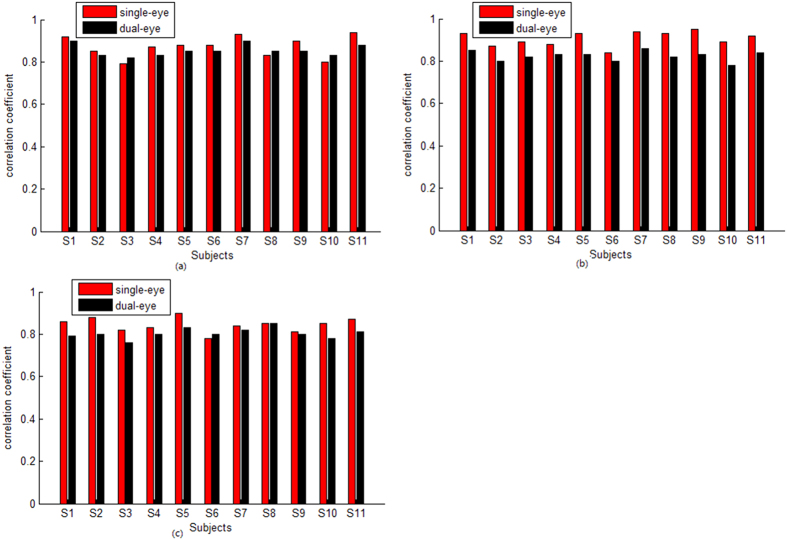
The correlation coefficient of the SSVEP distribution for all subjects under different stimulus style. The red bar indicates the correlation coefficient under the single-eye stimulus, while the black bar indicates that under the dual-eye stimulus. (**a**) indicates the correlation coefficient under the first stimulus style, (**b**) indicates the correlation coefficient under the second stimulus style, (**c**) indicates the correlation coefficient under the third stimulus style.

Under the other two stimulus styles, there were similar results with that under the first stimulus style. Figure 3 and 4 show the average SSVEP distribution across all subjects under the second and third stimulus style, respectively. Figure 5 shows the correlation coefficient of the SSVEP distribution for all subjects under different stimulus style.

## Discussion

Although the SSVEP has versatile uses, its mechanism is not clear yet. Here, we discussed its generation via the visual pathway. Light that is emitted from a small point source first projects on one or a few rod cells or cone cells in the retina. The electronic signals that are generated by these cells are then transferred to the ganglion cells and are pre-processed. The signal from a rod cell or cone cell corresponds to one or a few spots in space, while the signal from the ganglion cell does not correspond to specific spots in space. When the signal from ganglion cells is transferred to the primary visual cortex, the response of a neuron in the cortex cannot correspond to one or a few spots in space but rather represents the integrated signals in an area or even a whole visual field^21^,^22^,^23^,^24^,^25^. For a common visual stimulus, such as a picture, when a subject looks at it, the information including the luminance and color of every spot in space is projected onto the retina and then transferred to the primary visual cortex. This information is processed here first. Because this information has a unique meaning, it is then transferred to the high level area in the brain for further processing^3^,^26^,^27^,^28^,^29^. However, for a repetitive stimulus, such as a flicker, the luminance and color of every spot in the visual field are almost the same. Therefore, with the exception of a perceived twinkling, there is almost no other meaning to the visual stimulus. For this reason, we believe that further processing for this type of stimulus is limited, so the SSVEP power is primarily the result of the primary visual cortex with little contribution from any other brain areas.

In this work, the input to an SSVEP network is the same following single-eye stimulation or the simultaneous stimulation of both eyes with different frequencies. This is because the luminance and spatial structure of the point source is invariable under two conditions. However, the output of the primary visual cortex (i.e., the SSVEP) is different. Namely, the output following single-eye stimulation is significantly greater than following the simultaneous stimulation of both eyes. This suggests that a part of the SSVEP network is shared by another SSVEP network when the two networks are activated simultaneously. In other words, there is some physical overlap of these two networks. For example, only the 16.7 Hz SSVEP network is activated following stimulation in the right eye, while the 16.7 and 12.5 Hz SSVEP networks are activated simultaneously following the dual-eye stimulation under the first stimulation style. The input to the 16.7 Hz SSVEP network is the same under two conditions. If all the neurons in the 16.7 Hz network respond to the stimulus, the output of this network should be similar under two conditions. However, the result shows that the output of the 16.7 Hz SSVEP network following single-eye stimulation is bigger than that following dual-eye stimulation. This suggests a part of 16.7 Hz SSVEP network is shared by 12.5 Hz SSVEP network under situation of dual-eye stimulation. Namely, there is some overlap of the 16.7 and 12.5 Hz SSVEP networks.

There are several types of neurons in the cortex. Although their functions are different, the mechanism of generating and transferring electronic signals is approximately the same^30^,^31^. The signal transfer between neurons is based upon the synapse, and most synapses are chemical in nature. A neuron has many dendrites and one cell body, and the chemical signals from the synapse can be collected through the dendrite and cell body. These signals are integrated at the axon hillock, which is connected with the cell body, prior to being transferred to the next synapse through the axon. The generation of an electronic signal in the axon hillock has an ‘all or none’ response, which means a sub-threshold input will not produce an action potential. If the input is higher than the threshold, an action potential is generated while the intensity of the action potential is invariable, and the axon can transfer this signal to the next synapse without attenuation. This means the electronic signal in the axon can have the same intensity for any frequency of stimulus. Despite being common, chemical synapses consume variable amounts of time during information transfer. Due to the ‘all or none’ property of the axon hillock, if the next stimulus arrives within the response period of an axon hillock, it is neglected. This can result in variable frequency-amplitude characteristics of synapse for different frequency stimuli. According to some hypothesis the EEG is the measure of different potentials among the dendrites, but not the action potential resulted by the axon. Synchrony of firing of many neurons is necessary for the EEG observed from the scalp^30^,^31^. The dendrites collect the signals from the other axons through the synapse and the dendritic currents regulate the firing, then the signal transfers step by step. The SSVEP is a kind of EEG, its intensity depends on the dendritic currents which is related to its connected axons. Because the action potential of each axon has almost the same strength, the SSVEP intensity is related indirectly to the amount of the neurons that are involved. In response to different frequency stimuli, the SSVEP power is different, which suggests that a variable number of neurons are involved in accordance with the frequency of the stimulus. Namely, there are some differences between different SSVEP networks.

The signal transfer between neurons is often explained using low-pass filter theory^32^,^33^,^34^,^35^. The pass-band of different neurons and its associated structure can vary. This suggests that the number of neurons that are involved following various frequency stimuli can be different, which then results in a different intensity of SSVEP; however, only using this low-pass filter theory to explain the signal transfer between neurons has some limitations. If all neurons and their associated structures were viewed as low-pass filters, then this means that the neural networks that respond to the high frequency stimuli can all be involved in low frequency stimuli. So, the power of the low frequency SSVEP should be always higher than that of the high frequency SSVEP. However, this is not the case.

Due to the ‘all or none’ property of the axon hillock, if the stimulus frequency is high, then subsequent stimuli arrive during the refractory period of the last stimulus, resulting in an unresponsive axon hillock and the low-pass property of the signal transfer between two neurons. In chemical synapses, chemical material can decrease during the transfer period. If the stimulus frequency is too low, then the chemical material that is accepted by the next cell body and its dendrites cannot activate the axon hillock because of the low density that results from infrequent stimuli. This can result in a high-pass property of signal transfer in the synapse. In connecting the low-pass property of the axon hillock with the high-pass property of the synapse, the signal transferring between the neurons can have a property of band-pass filter. Due to different spatial structures and chemical substances, the pass-band of different neurons and their associated structure are different. These neurons and the associated structures distribute randomly and construct many signal channels. The input signal can go through any channel only if it can pass through all of the neurons in that channel.

Based on the band-pass filter theory of the signal channel, the number of involved neurons is different for varying frequency stimuli. Furthermore, although the response intensity of each neuron is almost the same, the SSVEP power for different frequency stimuli is different. Because of the band-pass property of the signal channels, different frequency stimuli can activate different neurons, thereby supporting the hypothesis that many SSVEP neural networks exist. Because the pass-band of these filters can overlap, the SSVEP neural networks can also overlap. The previous study has shown that there are three similar SSVEP response curves in different bands. While this curve can indicate that the amount of neurons that are involved under different frequency band stimuli is similar, it does not indicate that there are only three SSVEP networks.

When two SSVEP networks are activated under the condition of simultaneous stimulation of both eyes, the independent part of each network can function similar to the condition of single-eye stimulation. Based on the axon hillock’s ‘all or none’ property, the overlapping part would respond to the two stimuli alternately. Therefore, the average response for each stimulus by this overlapping part would be lower than following a single-eye stimulation. In this way, the SSVEP of a certain frequency following single-eye stimulation is higher than that following the simultaneous stimulation of both eyes.

## Methods

### Ethics Statement

The methods were conducted in accordance with the approved guidelines. This work was approved by the Human Research and Ethics Committee of University of Electronic Science and Technology of China. Before the experiments, all subjects were informed of the purpose and procedures of this experiment in detail and were asked to sign a consent form. These forms were sent to the Ethics Committee of our university, which provided approval for the conduct of this study.

### Stimulus design

Eleven subjects were involved in this experiment. They had normal or corrected normal acuity, with both eyes having the same acuity. Two cylinders of approximately 60 cm length were connected together to construct a double-barrel device such as a telescope, and the distance between the centers of the two cylinders was approximately 10 cm. Two high luminance focused LEDs were fixed at one end of the double-barrel device with one LED in each cylinder, and this end was then covered by an opaque paperboard. These two LEDs were driven by different frequency square waves separately or simultaneously and were utilized as the SSVEP stimulator of the left eye and right eye. Because the LEDs are focused and the subjects receive the light through a barrel, the stimulus can be considered as a point source. This kind of design can decrease the influence on the scalp topography by the spatial structure of the stimulus. The luminance of each LED is 2.5 cd/m^2^. During the experiment, the subjects looked at the flicker in the other end of the double-barrel device. When the two LEDs flickered simultaneously, the subjects could see one flicker in left eye and the other flicker in the right eye. When only one LED flickered, the subjects could see one flicker in a half-visual field, and the other half-visual field was dark. Prior to flicker stimulus, the spontaneous EEG was recorded for 40 seconds, which was then used to compute a baseline of a certain frequency. The experiment included three kinds of stimulus styles.

The stimulus frequencies were 12.5 Hz in the left eye and 16.7 Hz in the right eye in the first style stimulus. First, the 12.5 Hz flicker in the left eye flashed for 40 seconds independently; then the 16.7 Hz flicker in the right eye flashed for 40 seconds independently. Finally, these two flickers flashed for 40 seconds simultaneously. In each stage, the evoked EEG was recorded.

The stimulus frequencies were 12.5 Hz in the left eye and 8.3 Hz in the right eye in the second style stimulus. Then, the stimulus frequencies were.6.3 Hz in the left eye and 16.7 Hz in the right eye in the third style stimulus. The experiment steps under these two stimulus styles were similar to that of the first style.

### EEG recording

The recording was conducted using the “Net Amps 200” amplifier with a 129 channels electrode cap (EEG system 200, Electrical Geodesics Incorporated, USA). The 129 channels included 128 measuring electrodes and one reference electrode ‘Cz.’ The international 10–20 recording location was included in this system. Electrode impedance was maintained below 10 kΩ to maintain contact with the scalp. Salt water was dropped into the electrode periodically. The sample rate was 250 Hz, which meant the EEG would be sampled every 4 ms. The sampled signal was then filtered by a band-pass filter of 0.3–45 Hz and stored on a disk for off-line analysis.

### Data analysis

Because the SSVEP is relatively immune to low frequency noises, such as eye or body movement, no other pre-process method, such as the removing the eye movement, was adopted in the data analysis. The 40-s evoked EEG data at each electrode was processed by FFT directly. The relative-power of the SSVEP at all electrodes was computed and summed together as the SSVEP power under that stimulus. In order to compare the intensity of the SSVEP with the corresponding frequency component in the spontaneous EEG, the relative-power of a certain frequency in spontaneous EEG was computed in the same way as that in the evoked EEG. For a stimulus ‘f’ Hz, the relative-power of this frequency is computed as follows:


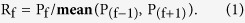


Where ‘R_f_’ represents the relative-power of ‘f’ Hz, ‘P_f_’ represents the absolute-power of ‘f’ Hz, ‘**mean**(P_(f−1)_, P_(f+1)_)’ represents the average absolute-power from ‘f−1’ Hz to ‘f + 1’ Hz.

In order to assess the difference significance of the SSVEP power following different stimuli, one-way Analysis of Variance (ANOVA) was performed under every condition. The SSVEP distribution can be understood via a series of 129 points. In order to understand the difference significance of the SSVEP distribution following varying stimuli, two-way ANOVA was used to check the SSVEP distribution series. The significance level ‘p’ was set to 0.05. If the ‘p’ was smaller than 0.05, then the difference between the two compared conditions was considered significant.

## Additional Information

**How to cite this article**: Wu, Z. Physical connections between different SSVEP neural networks. *Sci. Rep.* **6**, 22801; doi: 10.1038/srep22801 (2016).

## Figures and Tables

**Table 1:** Relative-power of SSVEP under different stimulus styles.

Stimulus style	Subject		S1	S2	S3	S4	S5	S6	S7	S8	S9	S10	S11	ANOVA(p)
First stimulus style	Relative power of 16.7 Hz	***single-eye***	519	437	425	485	325	281	279	387	260	299	300	0.01
		***dual-eye***	323	343	364	427	388	242	275	350	237	279	320	
	Relative power of 12.5 Hz	***single-eye***	477	435	440	523	546	281	308	389	255	235	298	0
		***dual-eye***	360	419	436	399	466	359	325	374	248	260	281	
Second stimulus style	Relative power of 12.5 Hz	***single-eye***	477	435	440	523	546	281	308	389	255	235	298	0.01
		***dual-eye***	396	382	362	326	479	263	334	456	245	230	349	
	Relative power of 8.3 Hz	***single-eye***	393	366	350	335	279	260	276	323	348	239	321	0.03
		***dual-eye***	392	346	270	363	394	260	243	300	359	247	256	
Third stimulus style	Relative power of 16.7 Hz	***single-eye***	519	437	425	485	325	281	279	387	260	299	300	0.03
		***dual-eye***	318	305	349	351	330	245	255	400	231	320	262	
	Relative power of 6.3 Hz	***single-eye***	381	363	323	293	346	297	369	278	315	258	252	0.02
		***dual-eye***	356	318	309	273	343	241	265	266	332	291	258	
